# Zebrafish—An Optimal Model in Experimental Oncology

**DOI:** 10.3390/molecules27134223

**Published:** 2022-06-30

**Authors:** Iwona Kwiatkowska, Justyna Magdalena Hermanowicz, Zaneta Iwinska, Krystyna Kowalczuk, Jolanta Iwanowska, Dariusz Pawlak

**Affiliations:** 1 Department of Pharmacodynamics, Medical University of Bialystok, Mickiewicza 2C, 15-222 Bialystok, Poland; justyna.hermanowicz@umb.edu.pl (J.M.H.); farmakodynamika@umb.edu.pl (Z.I.); jolanta.iwanowska1@umb.edu.pl (J.I.); dariusz.pawlak@umb.edu.pl (D.P.); 2 Department of Clinical Pharmacy, Medical University of Bialystok, Mickiewicza 2C, 15-222 Bialystok, Poland; 3 Department of Integrated Medical Care, Medical University of Bialystok, ul. M Skłodowskiej-Curie 7A, 15-096 Bialystok, Poland; krystyna.kowalczuk@umb.edu.pl

**Keywords:** zebrafish, oncological model, tumor microenvironment

## Abstract

A thorough understanding of cancer pathogenesis is a necessary step in the development of more effective and safer therapy. However, due to the complexity of the process and intricate interactions, studying tumor development is an extremely difficult and challenging task. In bringing this issue closer, different scientific models with various advancement levels are helpful. Cell cultures is a system that is too simple and does not allow for multidirectional research. On the other hand, rodent models, although commonly used, are burdened with several limitations. For this reason, new model organisms that will allow for the studying of carcinogenesis stages and factors reliably involved in them are urgently sought after. *Danio rerio*, an inconspicuous fish endowed with unique features, is gaining in importance in the world of scientific research. Including it in oncological research brings solutions to many challenges afflicting modern medicine. This article aims to illustrate the usefulness of *Danio rerio* as a model organism which turns out to be a powerful and unique tool for studying the stages of carcinogenesis and solving the hitherto incomprehensible processes that lead to the development of the disease.

## 1. Introduction

**Figure 1:**
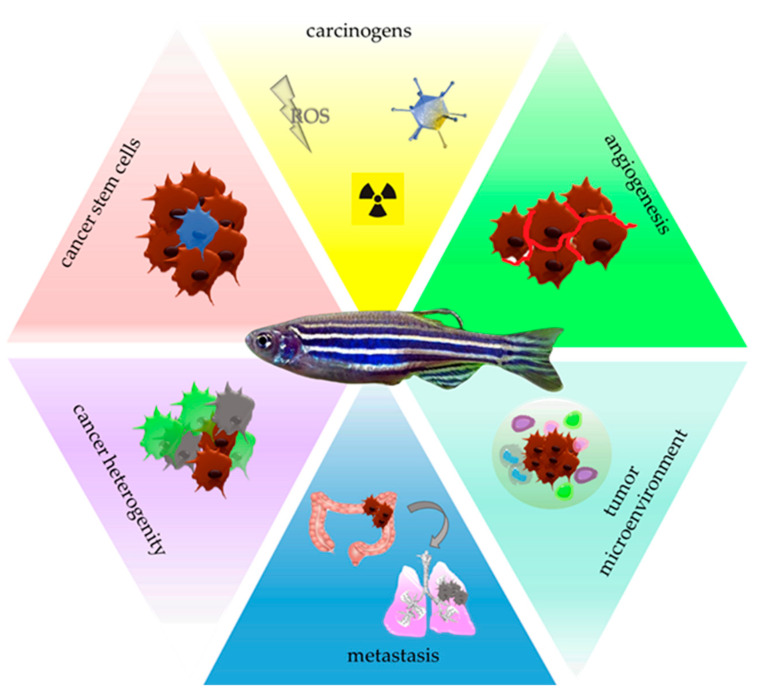
Zebrafish as a model to study individual stages of carcinogenesis. ROS—reactive oxygen species.

Cancer remains one of the leading causes of disease-related death throughout the whole world. Great efforts are taken to understand its biology and factors that contribute to tumor initiation, progression, and persisting failure of therapies. Despite advanced research, new trends in oncology are coming to light, and they include the development of new techniques and models, which will allow for an in-depth understanding of this disease as a result of intertwining phenomena and interactions, leading to the development of cancer in the human body. The most popular in oncological studies are rodent models; however, they are burdened with several limitations, which include high costs of research, long waiting time for offspring, and their limited number, as well as the fact that rodents are furry, which makes it impossible to visualize processes in a real-time manner. Due to that fact, alternative organisms which could serve as a reliable model are highly sought after. The animal that in recent years has gained great scientific interest in translational studies is a small sweetwater fish—*Danio rerio*, also known as the zebrafish. This inconspicuous vertebrate is endowed with many advantages that allow it to be included in research, enabling a better understanding of the malignancies (Figure 1). 

Zebrafish have high genetic homology with the human body. The sequenced genome (*Danio* has 25 chromosomes) and the presence of approx. 70% of human genes make this animal unique [1,2]. Major organs and tissues (brain, heart, kidneys, muscles, liver) share many features with humans, both anatomically, physiologically, and molecularly [3,4,5,6]. The easiness of reproduction and a short development cycle (approx. 3 months from egg to adult), the possibility of frequent obtaining large amounts of research material, the simplicity of genetic manipulation, and the abundance of mutants and the transgenic lines collection are undoubtedly some of the most important aspects, giving zebrafish an advantage over mouse or rat research models. Zebrafish mature within about three months after fertilization. This allows the conducting of experiments with high throughput, with the use of a small number of reagents, low financial costs, and better statistical evaluation of the obtained results (large research groups). Equally important is the fact that in the first days of life, zebrafish embryos are relatively large and transparent, which makes it possible to observe and document processes taking place in the body in real-time [7]. What is more, the *Danio rerio* immune system retains a lot of homology with the human system, which is an invaluable tool for researching the newly-growing field of science—immuno-oncology [8]. All this makes the zebrafish an indispensable tool to face the challenges posed by modern oncology in terms of understanding cancer pathogenesis. This article aims to present *Danio rerio* as a unique organism that can be used as a complementary model to those commonly used ones, and which may help to overcome an existing limitation in oncological research. The knowledge gained from research using this model organism will certainly contribute to a better understanding of cancer development and establishing new treatment strategies. 

## 2. Zebrafish—A Tool for Carcinogens Screening

According to the International Agency for Research on Cancer, carcinogens are divided into 10 groups based on their mechanisms of action: (1) substances that act as an electrophile; (2) genotoxic agents; (3) factors that cause genomic instability and impair DNA repair; (4) inductors of epigenetic alterations; (5) oxidative stress generators; (6) inductors of chronic inflammation; (7) immunosuppressants; (8) modulators of receptor-mediated effects; (9) factors which cause immortalization; and (10) those which alter cell proliferation, cell death, or nutrient supply [9]. The identification of potentially dangerous factors and discovering their mechanism of action allow to define risk groups and establish new methods of cancer prevention. Conducting screening tests with the use of *Danio rerio* allows for the identification of oncogenic molecules and the detection of occurring changes leading to cancer initiation [10]. For example, up-to-date genotoxicity is evaluated by the micronucleus (MN) assay. In short, an MN is a part of the main nucleus and serves as a marker of chromosomal damages [11]. Le Bihanic et al. showed that the exposition of zebrafish to different genotoxic substances (mitomycin C, etoposide, cyclophosphamide, demecolcine, benzo[a] pyrene, and dibenzo chrysene) induces the creation of MNs in this animal, which points to the usefulness of *Danio rerio* in testing if any agents bear genotoxic potential [12]. Other methods used to test a substance’s genotoxic potential are AFLP (amplified fragment length polymorphism) and qRAPD (quantitative random amplified polymorphic DNA), and both of them detect DNA impairment. Srut et al. exposed *Danio rerio* larvae and adults on benzo[*a*]pyrene and ethyl methanesulfonate and evaluated DNA changes using the aforementioned methods [13]. The authors point to the high sensitivity of *Danio rerio* as a biosensor of genotoxicity with the use of these methods. This not only emphasizes the usefulness of this organism in the imaging DNA impairment but also shows that multiple methods can be applied, which extend the role of the fish in tracking genotoxic substances. Occurring DNA damages are repaired by one of the few pathways, among which mismatch repair (MMR) is the most common one. Deregulation of this pathway is one of the hallmarks of cancer, and such carcinogens as cadmium and benzo[a]pyrene (BaP) are known factors impairing the MMR process, so they are used to validate the sensitivity of the model to occurring disturbances. *Danio rerio* model created by Chen et al. allows for the observation of impairments in this essential for genome stability process [14]. The method is based on the quantification of the expression of the enhanced green fluorescent protein (EGFP) gene in MMR-competent zebrafish and its reduction in animals lacking this mechanism. The reduction of EGFP gene expression in individuals exposed to MMR-impairing factors proves that zebrafish is a valid sensor for detecting disruption in the DNA repair process, which occurs under the impact of environmental pollutants. Oxidative stress and reactive oxygen species (ROS) are the next well-known risk factors leading to cancer promotion. The recently created transgenic zebrafish line (*3EpRE:hsp70:mCherry*) shows high sensitivity to RedOx imbalance and enables visualization of tissue-specific changes after exposure to drugs and chemicals [15]. The authors of this experiment checked five ROS-generating compounds (diethylmaleate, acetaminophen, cisplatin, phenylhydrazine, and Cu^2+^) and illustrated tissue-specific RedOx changes, validating the same use of the zebrafish in detecting increasing oxidative stress and the most vulnerable organs on occurring cancerous changes. Oxidative stress is inextricably linked with chronic inflammation—another hallmark of cancer [16]. Individual components of the inflammatory response, triggers, mediators, and the involved immune cells can be studied in the *Danio rerio* model. For this purpose, zebrafish xenografts, i.e., organisms with an implemented human tumor tissues, may be applied [17,18]. The experiment on zebrafish xenografts with hepatocellular carcinoma cells identified several chemicals, such as chromium, dioxins, and the organic toxicant PCB126 (a 3,3′,4,4′,5-pentachlorobiphenyl, aromatic hydrocarbon, and environmental pollutant) to cause increased neutrophils influx into the liver and its oncogenic growth. The tested arsenic impacted oncogenic liver growth, but at the same time, it decreased the number of neutrophils in the liver, while the organic toxicant TCDD (2,3,7,8-tetrachlorodibenzo-p-dioxin, one of the most potent carcinogenic environmental pollutants) decreased both the liver size and neutrophils influx [19]. Available pieces of evidence show the connection between environmental pollution and breast cancer [20,21], colorectal cancer [22,23], lung cancer [24], kidney cancer [25,26], and gastric cancer [27], so in future research zebrafish could serve as a model organism helping to define risk factors that lead to oncogenic transformation. Not only chemicals lead to the induction of oxidative stress and chronic inflammation. Viruses, such as HPV, HCV, HBV, and EBV, are also linked to these processes and are known agents that contribute to cancerogenesis [28,29,30,31]. In this regard, the zebrafish allows the studying of virus-induced oncogenesis. The co-expression of hepatitis B virus X (HBx) and hepatitis C virus core (HCP) proteins in zebrafish liver led to the formation of intrahepatic cholecarcinoma (ICC), proving the oncogenic potential of these viruses in *Danio rerio*. Moreover, in the course of the experiment, the authors were able to detect ICC markers and point to the induction of oncogenic pathways, such as MAPK, ERK1/2, and TGF-B, under the impact of viruses proteins [32]. This opens doors for future research focusing on virus-cause cancer promotion in the zebrafish model, which will help to discover its role in human pathogenesis. To summarize, data show the broad possibilities given by the inclusion of *Danio rerio* in research, focusing on carcinogens and other factors leading to cancer promotion. 

## 3. Zebrafish—A Tool for Studying Cancer Stem Cells

**Figure 2:**
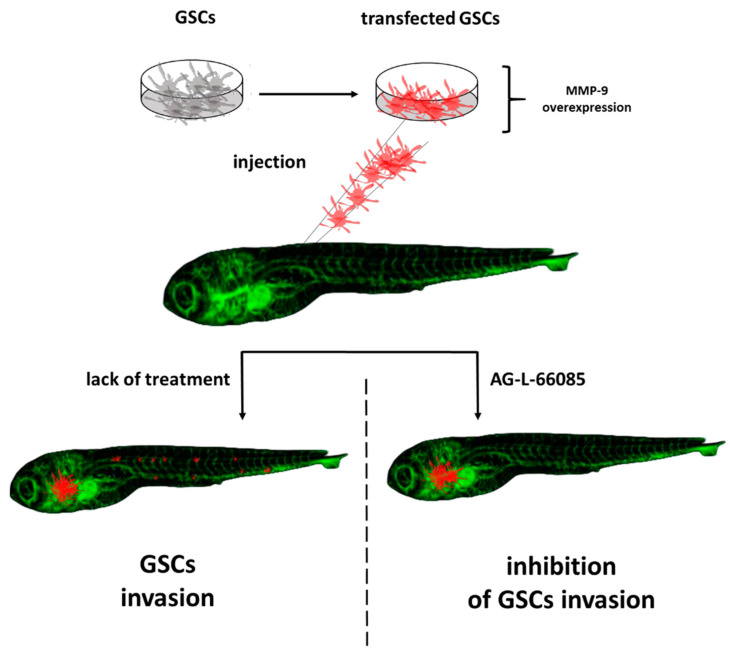
Participation glioma stem cells (GSCs) in the invasion using the zebrafish model. MMP-9-matrix metalloproteinase; AG-L-66085-MMP-9 inhibitor (based on results shown by Yang X jun et al.) [41].

In addition to the known carcinogens, the onset of cancer disease is searched for in so-called cancer stem cells (CSCs), which are endowed with self-renewal ability, differentiation capacity, and tumorgenicity [33]. It is suspected that CSCs are involved in further stages of oncogenesis and that they contribute to cancer progression, metastasis, chemoresistance, and therapy failure, but mechanisms leading to these outcomes remain an unresolved issue [34]. Additionally, the origin of CSCs is not clear. The existing hypotheses point to a fusion of cells with hematopoietic stem cells, horizontal gene transfer, genomic alternation and instability, and selective clonal expansion under the impact of the microenvironment as those factors contribute to the conversion of non-CSCs into CSCs [14]. The available methods to study CSCs behavior and their role in cancer development include a transplantation assay and a lineage-tracing assay. The first one is based on the xenotransplantation of CSCs into immunocompromised mice. The essence of the second test is a single cell marking in a way that allows for transmitting the mark to the cell’s progeny [35,36,37]. That results in the creation of labeled clone sets. These models are not ideal and are burdened with some problems. One of them is the difficulty of observation at the very beginning of the disease when the number of CSCs is low. Moreover, mouse models do not allow for real-time observation. The transparency of the zebrafish embryo is a decisive advantage in this context and provides a previously not reached possibility of observing single-cell behavior in a spatiotemporal manner. Chen et al. transplanted prostate CSCs labeled with red fluorescent protein into the *Danio rerio* transgenic line with green vasculature [38]. Thanks to this approach they were able not only to visualize CSCs dissemination but also to study the interaction between this subpopulation and vasculature. The authors of this experiment showed that CSCs have a greater ability to extravasate than non-CSCs and pointed to a strong involvement of macrophages and neutrophils in the process of neovascularization. The applied method used in that experiment uses a specific marker (ALDH^high^ and ALDH^low^), to distinguish between both types of cells. This approach was used to test the efficiency of CSCs-targeting therapy with the use of docetaxel and showed this drug as an effective CSCs proliferation inhibitor [39]. Similarly, Yang et al. point to another substance—gomisin M2—which was tested as a CSCs proliferation inhibitor in the *Danio rerio* breast cancers stem cells model [40]. The CSCs subpopulation was connected with driving metastasis. The zebrafish model with injected glioblastoma cancer cells developed by Yang served as a tool to identify MMP-9 as the major factor of increased invasiveness of these cells. In the same study, the authors confirmed the effectiveness of the MMP9 inhibitor in hindering the spread of cancer cells (Figure 2) [41]. 

## 4. Zebrafish—A Tool for Studying Angiogenesis

**Figure 3:**
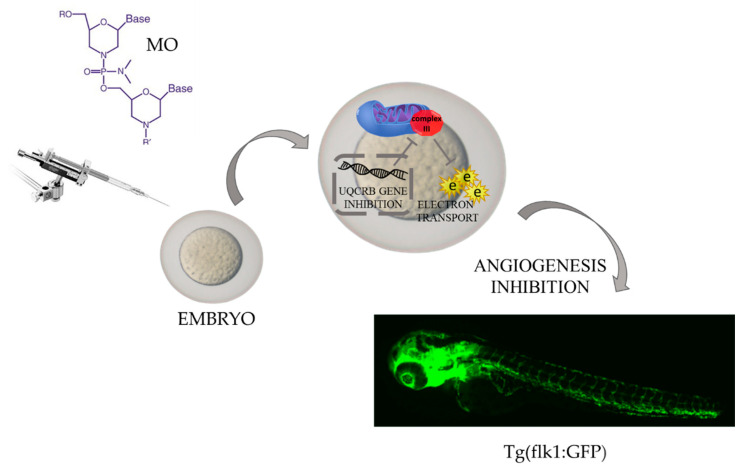
Oligonucleotide-based tools for studying gene function in zebrafish. The role of the gene-encoding ubiquinol cytochrome c reductase binding protein (UQCRB) in angiogenesis. E—electron, MO—morpholino oligonucleotides; Tg{flk1:GFP)—transgenic zebrafish line; UQCRB—ubiquinol–cytochrome c reductase binding protein (the mitochondrial complex III) (based on results shown by Cho YS et al.) [62].

Enhanced angiogenesis, which is controlled by many factors, such as the VEGF family [42], FGF family [43], platelet-derived growth factors (PDGFs) family [44], angiopoietins [45], chemokines [46], metalloproteinases [47], TNF-a [48], and inflammatory cytokines [49], remains a process that is not fully understood. Additionally, hypoxia and immune cells, which are inductors of pro-angiogenic signaling pathways [50,51], make the whole process multifactorial and provide a basis for asking questions about the tumor microenvironment’s role in inducing angiogenesis, interactions between cancer and vessel cells, and overlapping events that drive vessel formation. This complexity and unresolved issues may be a reason for insufficient efficacy in treatment with the use of anti-angiogenic drugs. With the use of *Danio rerio*, a few techniques for the visualization of angiogenesis were developed. Established transgenic fluorescent lines with green vasculature allowed for the visualization of ongoing vessel formation and the occurring alteration in a real-time manner. Up to date, two lines (fli1a:EGFP and kdrl:EGFP) are the most commonly used in angiogenesis research, and with their use, it is possible to image interactions between tumor cells and their microenvironment [52,53]. For example, in a TGF-B-pretreated glioma xenograft, Yang et al. observed vessel formation in a tumor cell number-dependent manner, and—which is highly valuable—the authors visualized macrophage infiltration into the angiogenic region, pointing to their involvement in the process [54]. What is more, it was proven that the proangiogenic role of macrophages varies according to the VEGFA level, in such way that they drive vessel formation under the upraised level of the mentioned factor [55]. Another study showed that inhibition of VEGFR, the next proangiogenic agent, impairs macrophage recruitment and ongoing angiogenesis [56]. Coming back to the issue of the induction of angiogenesis by tumor cells, it is important to mention that zebrafish in hepatoma xenografts led to point to WNK1 (with-no-lysine kinase 1) signaling as the main factor enhancing vessel formation [57]. Moreover, it is possible to visualize blood vessels in non-living zebrafish, where in situ hybridization and alkaline phosphatase staining are used for this purpose. The easiness of vessel visualization opens the door for investigating the broad spectrum of mechanisms leads to vessel formation. Zebrafish yolk membrane (ZFYM) assay allows for the observation of subintestinal vein (SIV) formation after the exposure to angiogenesis modulators. The confirmation of the practical application of this test may be the results of studies in which caffeine was identified as an anti-angiogenic compound, which exerts this effect both directly and by blocking FGF-signaling [58]. Zebrafish as a model organism allows for the creation of genetic modifications using the morpholino oligonucleotide (MO) injection method. It is based on knocking down specific genes to assess their role in a studied process and is widely used in learning about the cancer biology and drug discoveries [59,60,61]. Angiogenesis is one of the processes which may be studied thanks to this technique. Obtained through the MO injection method, the organism with the knock-out gene expression becomes a remarkable tool to study the role of a given gene in a physiologically occurring angiogenesis or tumor-induced one and to foster the establishment of signaling pathways induced by pro-angiogenic factors [52]. The application of MO led to a discovery that the gene-encoding UQCRB–mitochondrial complex III involved in electron transport is involved in the creation of angiogenic sprouts [62]. This shed light on mitochondria as organelles involved in vessel formation and points to the need for further oncological research to focus on this cellular structure (Figure 3). 

Another example of a pro-angiogenic protein identified in the *Danio rerio* model was protein kinase D isoenzyme 1 (PKD1), which, after knockout, abolished both physiologically occurring angiogenesis and the one ongoing in HCT116 xenograft [63]. This means that that the aforementioned kinase is involved in pathologically occurring vessel formation.

Aside from the abovementioned agents, the role of hypoxia as a proangiogenic factor, should be emphasized here. The state of insufficient oxygen levels is regulated by several factors, from which the HIF family is studied at the highest level. Nevertheless, the exact cascade of events induced by HIF is still not established and new aspects constantly come to light. For example, a recent finding shows that hypoxia-dependent pathways have an impact on immune cells—they drive their polarization into a pro-cancerous state and change cancer cell metabolism [64,65,66,67]. Due to the complexity of the processes, in vitro studies do not mimic the tumor microenvironment at a satisfactory level. Even more advanced 3D cell culture models are too simple to detect all existing cells interactions. On the other hand, experiments in rodents pose many problems. To investigate physically occurring hypoxia, animals need to be kept in specially adapted chambers, which is problematic when it comes to feeding and caring for animals. In the case of tumor-induced hypoxia, there is a problem with detecting hypoxic cells within the tumor mass. Zebrafish in this context gives a great opportunity to investigate processes induced by the lowered level of oxygen in the tumor microenvironment. First of all, they are not fed until 5 days post-fertilization, which allows them to be kept in the hypoxic chamber without disturbances for a longer period than rodents [68]. An undeniable advantage of *Danio rerio* is the possibility of imaging hypoxic cells in the living organism. Wang et al. showed that it is possible to monitor hypoxic cells in zebrafish embryos, with the use of an iridium(III) complex, which in hypoxic subpopulation undergoes reduction, with concomitant luminescence [69]. That gives the possibility for future studies investigating hypoxia modification under different conditions. The search for the pathways potentially involved in driving hypoxia led to a discovery that there is a cross-talk between glucocorticoid signaling and hypoxia, and the zebrafish model helped to establish that upraised HIF activity leads to decreased activity of the gluthecorticoid receptor and the mineralocorticoid receptor, but on the other hand, both receptors enhance HIF signaling [70]. Corticoids activity brings to mind their role in mediating the inflammatory response, and thus the activity of the immune cells. In zebrafish, hypoxia was shown as a factor inducing TNF-a expression on macrophages in a COX-dependent manner or neutrophils infiltration by myc-induced liver tumorigenesis [71,72]. These results show that zebrafish, thanks to the possibility of conducting multifaceted experiments, may be a unique tool for studying processes that lead to enhanced vessel formation around the tumor mass.

## 5. Zebrafish—A Tool for Investigating Circulating Tumor Cells

The metastasis cascade is a multi-stage and complex process. At the beginning, the intercellular connections in the primary tumor are loosened, then they penetrate inside the blood vessels in the process called intravasation, and then after circulating, along with the morphotic elements, tumor cells exit (extravasate) from the vessel to a new place of growth. What is important is that after intravasation tumor cells constitute a subpopulation of circulating tumor cells (CTCs), which differs from the primary tumor mass. These differences are seen since varied cellular markers and phenotypes allow them to survive in the bloodstream. Obviously, to fully understand their behavior, CTCs have to be investigated in a living organism. Present limitations of studying CTCs include insufficient detection in the patient’s blood samples, lack of possibility of monitoring very early metastasis formation in mouse models, insufficient knowledge about markers to capture different CTCs subpopulations, and a gap in the understanding of exact processes leading to intravasation and later endothelial adhesion, which conditions final metastasis formation. The zebrafish, thanks to features such as body transparency, seems to face these challenges and allows for the observation of CTCs behavior in a living organism in a previously impossible way. Follain et al. established a *Danio rerio* model, which allows an image of a single CTC and studied its behavior and role in micrometastasis formation in a spatiotemporal manner [73]. This more detailed study led to the discovery that reduced blood flow is a favorable factor for CTCs endothelial adhesion and results in an extravasation and metastasis formation [74]. Experiments with use of zebrafish also allowed for establishing the novel mechanism of extravasation, called angiopellosis—a process in which extravasating cells retain their original shape and the driving forces are vascular endothelial cells, which remain the most active throughout the process [75]. It seems that cancer cells can extravasate in this manner as both clusters and individual cells, which is particularly important in regards to the report pointing to CTCs that enter the bloodstream in clusters, like the ones with higher survival ability and bigger metastasis potential when compared to single circulating cells [76]. It was observed in the melanoma zebrafish model that cells that exit as a cluster form bigger tumor masses than those that existed as single cells [77,78]. It was also previously reported that CTCs clusters are characterized by maintained stemness phenotype, higher surveillance, and capacity for immune escape [79]. The possibility for their examination in the zebrafish model may advance discoveries in this field and the development of new therapeutic options targeting CTCs. Regarding pharmacotherapy, it is worth mentioning that the *Danio rerio* model for testing personalized therapy has been already used by Fieuws et al., who xenografted patient-derived ovarian cells into a zebrafish embryo and tested different therapeutic options to establish the most effective one [80]. Despite the fact that it is not yet a widespread or repeatedly used method, the published report opens up new perspectives in research on individualization of the treatment. These initial findings indicate a great potential of *Dario rerio* to extensively investigate CTC biology.

## 6. Zebrafish—A Tool for Studying the Tumor Microenvironment

**Figure 4:**
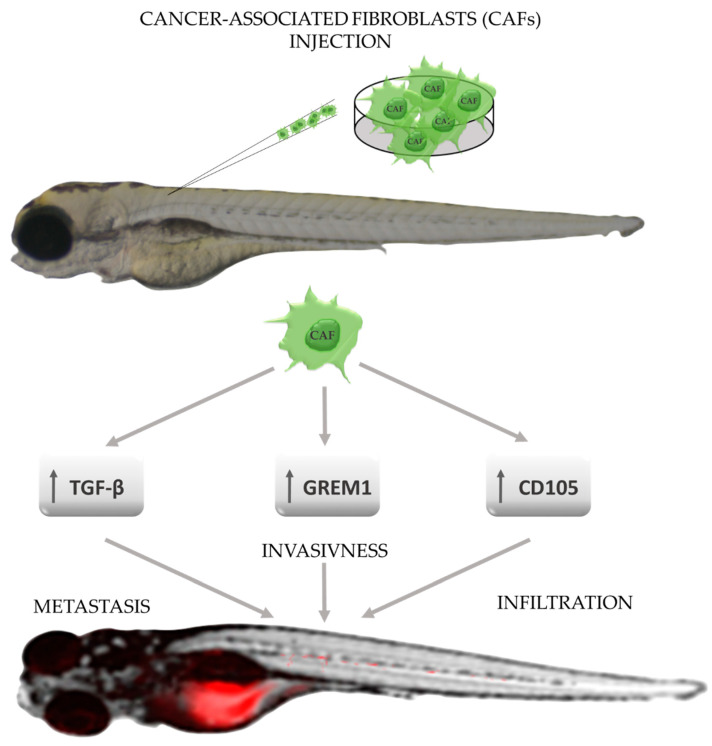
The role of cancer-associated fibroblasts (CAFs) in promoting tumor progression in zebrafish xenografts. CAFs—cancer-associated fibroblasts; CD105—endoglin, homodimeric transmembrane glycoprotein belongs to TGF-β family receptors, GREM1—gremlin 1, bone morphogenic protein antagonist; TGF-β—transforming growth factor β (based on results shown by Sun DY et al., Ren J et al., and Paauwe M et al.) [84,85,86].

Another element of the cancer machinery that highly conditions disease progression is the tumor microenvironment (TME). The elements which constitute TME can be in general divided into three main groups—infiltrating immune cells, angiogenic vascular cells, and cancer-associated fibroblasts (CAFs) [81]. The latter is suspected to take part in the proliferation and survival, angiogenesis, metastasis, immunogenicity, and resistance to therapies [82]. However, their origin, phenotypes, and exact role in individual disease’s stages remain undiscovered. As in the case of CTCs, the zebrafish is a great tool to study the role of CAFs through the perspective of a single cell, which creates a possibility to evaluate the behavior of CAFs and metastasis formation from its beginning, which significantly raises the chances of developing new anti-metastatic drugs. The possibility of conducting research with the use of labeled CAFs has led to the discovery of their role in enhancing the invasiveness of cancer cells and to the observation of the phenomenon of close adherence of migrating cancer cells to CAFs [83]. The zebrafish model also provides a platform for revealing mechanisms and factors, which drive their enhanced invasiveness and metastasis potential, and thus TGF-B, GREM1, and endoglin have been identified as major process mediators and may become targets for new therapies [84,85,86] (Figure 4).

It should be noted that the abovementioned experiments were conducted with the use of different cancer types of cell lines, which makes zebrafish a versatile model for further broad research.

Infiltrating immune cells are meaningful TME elements that modify cancer biology. All types of immune cells can be found in the tumor microenvironment, but their exact role as cancer modulators remains unresolved and needs to be investigated in detail together with the use of zebrafish as a model in this field. Leukocytes—macrophages or neutrophils—are suspected to exert a trophic, i.e., maintaining life function, effect on pre-neoplastic cells (PNCs). It was shown that PNCs secrete chemoattractant, which induce the migration of leukocytes. That results in enhanced PNCs proliferation and survival. Cancer modeling in zebrafish allows for the identification of key factors modulating leukocytes recruitment, such as H2O2, TGF-B, and CSF-1, which may become therapeutic targets in a novel immune-based therapy [87]. With the use of the *Danio rerio* model it was shown that neutrophils influx into the tumor environment correlates with increased vessel formation and macrophages polarization, pointing to the utility of zebrafish in investigating interactions between immune cells [88]. The involvement of neutrophils in cancer progression remains a puzzle, especially since outcomes from patient-derived samples are ambiguous and neutrophils infiltration is considered to be either a poor prognosis factor or a favorable one depending on the cancer type [89,90,91]. The zebrafish transgenic Tg(mpx: GFP)^I^ line in which neutrophils express a green fluorescent protein has already been established, and with its use researchers have indicated that this cell line is a great source of CXCR4—a receptor connected with cancer progression [92,93]. Furthermore, this protein was described as both a factor conditioning neutrophil motility and a modulator of early metastasis formation in different cancer cell lines [94]. As mentioned, neutrophils drive macrophage polarization, and similar effects are exerted by tumor metabolites and transcription factors activated by them [95,96]. In this regard, it is essential to highlight that both subsets of macrophages (anti-cancerous M1 and pro-cancerous M2) may be labeled in zebrafish, which broaden the utility of the model organism for screening immunomodulating agents [97]. The exact role of macrophages in cancerogenesis can be visualized and studied in zebrafish, which was reported by Britto et al., who showed the intense involvement of macrophages in vessel formation in a VEGFA-dependent manner [55]. This was possible through the establishment of the zebrafish model with a knocked out VEGFA ortholog (vegfaa), which may be included in another study focusing on the role of this factor in cancer progression. The zebrafish model with labeled macrophage led to the discovery that those immune cells, after stimulation by pro-inflammatory factors, such as IL-6 and TNF-a, enhance metastasis formation, and, what is more, metastatic tumor cells remain coupled with them [98]. As previously seen, the involvement of the *Danio rerio* organism in cancer research facilitates the acquisition of groundbreaking data which may significantly accelerate cancer research at many levels. As mentioned at the begging of this subsection, other immune cells are present in the tumor microenvironment as well. Their pro- or anti-cancerous role needs to be investigated in detail, and the fact that T-cells, B-cells, eosinophils, and mast cells are present in the organism of Zebrafish opens up a wide range of possibilities for future oncology research [99]. Additionally, it is important to remember that there is a big gap between the development of innate and adaptive immune mechanisms in zebrafish. Until the 6th week of development, the main defensive role is played by innate immune systems [100]. Only after this point do the mechanisms of adaptive response develop, which makes it possible to study the selective role of innate response mechanisms in cancerogenesis. 

## 7. Zebrafish—A Tool for Facing Other Challenges of Modern Oncology 

Another challenge, which modern oncology should face in the context of effective therapy is the heterogeneity of cancers, is the fact that genetic and metabolic differences between cancer cells within the same tumor mass has a great influence on the tumor growth and progression and determines the success of the therapy [101,102]. This aspect has not been yet studied broadly with the use of the *Danio rerio* model; however, there was a reported experiment in which triple transgenic zebrafish allowed for the labeling of tumor cells in stages [103]. Later, the model served to investigate the interaction between heterogenic tumor cells and zebrafish vasculature [104]. This model is gaining growing importance in the study of tumor heterogeneity. 

The problem of adult cancer is commonly known and has been broadly studied. Nevertheless, this disease does not only affect grown-up patients. The pediatric population is exposed to cancer development as well, although cancers most commonly occurring in children significantly differ from those in adults. Different genetic mechanisms and relatively rare occurrences are the main problems that make it difficult to understand the biology of pediatric cancers. Acute lymphoblastic leukemia (ALL) is among the most common pediatric tumors, and for this malignancy, zebrafish models have been developed [105]. A platform created through this method was used to assess genes’ role in ALL development and to perform drugs screening. For example, a study that aimed to validate the role of the MYC oncogene in the pathogenesis of this disease has led to the creation of a fish model, which provides the previously unattainable possibility of studying B-ALL and T-ALL in one organism [106]. The results of another study that focused on the role of MYC in ALL pointed to this gene as the one involved in developing cancer cell resistance to steroid treatment. The next gene, whose role in ALL pathogenesis has been confirmed by the use of *Danio rerio* is LDHA, delayed the disease development through its knockdown [107]. The gathered information only briefly outlines the possibilities of *Danio rerio* in pediatric cancer research. Detailed data on the issue of the use of zebrafish in pediatric cancers can be found in the study published by Caysey et al., to which we refer readers, not wanting to duplicate the messages contained therein [108]. Therefore, that information will not be repeated here.

## 8. Conclusions

Every year oncological research brings to light the answers to many questions concerning the nature of cancer. However, there is still an enormous amount of information, correlation, and interaction that has to be discovered to efficiently treat these malignancies. With each discovery, it becomes all the more clear that cancer cannot be regarded as a disease of one type of cell, tissue, or organ. It seems that the entire body is involved in the development of this disease, and the intertwining events should be studied inseparably. In this multitude of hitherto incomprehensible processes, a small fish with great scientific power appears to be an extremely helpful tool in finding the answer to the never before resolved issues which still pose a great challenge for modern oncology.

## Data Availability

The study not report any data.

## Sample Availability

Samples of the compounds Not available are available from the author.
